# A Community-Driven Framework to Prioritize the Use of Donated Human Biological Materials in the Context of HIV Cure-Related Research at the End of Life

**DOI:** 10.20411/pai.v8i1.583

**Published:** 2023-05-24

**Authors:** Karine Dubé, Thomas J. Villa, Jeff Taylor, Andy Kaytes, David J. Moore, Susan J. Little, Antoine Chaillon, Davey M. Smith, Sara Gianella

**Affiliations:** 1 Division of Infectious Diseases and Global Public Health, School of Medicine, University of California San Diego, La Jolla, CA; 2 AntiViral Research Center, University of California San Diego, San Diego, CA; 3 Health Policy and Management, UNC Gillings School of Global Public Health, Chapel Hill, NC; 4 HIV Obstruction by Programmed Epigenetics Delaney Collaboratory Community Team, San Francisco, CA; 5 Reversing Immune Dysfunction Delaney Collaboratory for HIV Cure Research Community Advisory Board, La Jolla, CA; 6 HIV + Aging Research Project – Palm Springs, Palm Springs, CA; 7 AntiViral Research Center Community Advisory Board, University of California San Diego, San Diego, CA; 8 Department of Psychiatry, School of Medicine, University of California San Diego, San Diego, CA

**Keywords:** End of life, HIV cure research, ethics, human biological materials, tissues, donations, pathogens, virology, immunity

## Abstract

Initiated in 2017 after extensive community engagement, the Last Gift program enrolls altruistic volunteers willing to donate their cells and tissues at the end of life to allow studies on HIV reservoir dynamics across anatomical sites. As the Last Gift team received tissue requests outside the scope of HIV cure research, we noticed the absence of guiding frameworks to help prioritize the use of altruistically donated human biological materials. In this commentary, we present a proposed framework for prioritizing the use of donated human biological materials within and outside the end-of-life (EOL) HIV cure research context, using the Last Gift study as an example. First, we discuss regulatory and policy considerations, and highlight key ethical values to guide prioritization decisions. Second, we present our prioritization framework and share some of our experiences prioritizing requests for donated human biological materials within and outside EOL HIV cure research.

## INTRODUCTION

The advent of modern antiretroviral therapy (ART) has transformed HIV from a terminal disease to a manageable chronic condition for most people with HIV (PWH). Over 40 years into the HIV epidemic, most PWH have a near-normal life expectancy and now die from non-AIDS defining illnesses resembling those of the general population, such as cardiovascular diseases, solid cancers, or neurodegenerative conditions [1–3]. Several long-term survivors of HIV have expressed a strong desire to give back to scientific research to benefit future generations [4].

In particular, PWH want to give back to science by donating their organs and tissues to help advance the search towards HIV cure [4–7]. For example, the Last Gift is an observational cohort study at the University of California, San Diego (UCSD) that evaluates HIV persistence throughout the entire body of PWH [8, 9]. Initiated in 2017 after extensive community engagement, the Last Gift program relies on altruistic volunteers willing to donate their cells and tissues at the end of life (EOL) to allow studies on HIV reservoir dynamics across more than 40 anatomical sites. Because HIV materials begin to deteriorate immediately after death [5], the autopsy must be performed within 6 hours post-mortem (called *rapid research autopsy*) [8, 10]. This differs from routine non-research autopsies conducted to determine the cause of death. The Last Gift has become a well sought-after resource for cutting-edge HIV research, which often goes beyond HIV cure efforts. For example, tissues from Last Gift participants have been requested to contribute to research on the topics of aging, cardiovascular and pulmonary diseases, and cancer.

The intent of the Last Gift is for PWH to donate cells and tissues to advance observational HIV cure research. At this juncture, the Last Gift is facing a new ethical conundrum: prioritizing donations of human biological materials (eg, cells, tissues, organs) for research use outside the HIV cure scientific context (initial intent). On occasion, the Last Gift team is asked by external investigators for requests of human biological materials for non-HIV cure-related research. Approximately 10% of Last Gift participants themselves have also asked informally for tissue donations outside the scope of HIV cure research to maximize their contributions to science and society.

Using human biological materials for non-HIV cure-related requests calls for a clear prioritization framework [11]. This is necessary because of limited resources both in terms of tissue types (for example, lymph nodes, or small brain structures are highly requested but have limited availability) and also laboratory personnel to execute the requests, which are often time-consuming. As the Last Gift team received requests for non-HIV cure research, we noticed the absence of guiding frameworks to help prioritize the use of donated human biological materials. We found that the conundrum of prioritizing multiple uses of human biological materials similarly arose in the context of cancer research at the EOL [11].

In this article, we present a proposed framework for prioritizing the use of donated human biological materials within and outside EOL HIV cure research, using the Last Gift study as an example. Our framework was entirely driven by members of the community, who advised and guided the Last Gift program at UCSD. First, we discuss regulatory and policy considerations and highlight key ethical values to guide prioritization decisions. Second, we present our prioritization framework and share some of our experiences and successful outcomes prioritizing requests for donated human biological materials within and outside EOL HIV cure research. We share our lessons learned in the hopes that these may help guide other research teams conducting rapid research autopsies facing similar decisions and set clear rules and expectations in a proactive way.

## REGULATORY AND POLICY BACKGROUND

There are extensive laws, regulations, and policies that govern research with human participants [12]. In the United States, research institutions observe the Federal Policy for the Protection of Human Subjects, also known as the Common Rule [13]. The Common Rule explicitly defines human research participants as living individuals [14]. Given that the Last Gift recruits PWH at the EOL, the program falls under the Common Rule requirements for Institutional Review Board (IRB) oversight. Investigators must obtain from each participant informed consent that includes the required elements specified per the Common Rule [14]. An important nuance, however, is that IRBs have no legally mandated regulatory oversight over deceased donors or donated human biological materials [14]. Under federal law, research involving deceased persons generally is not considered research with human participants and does not require IRB review and approval. However, there are some exceptions, for example, if the data or samples contain (or are linked to) any personal identifiers or if the information collected will obtain information relevant to any living relatives (eg, genetic studies). As regulations and exceptions might change by state, we recommend consulting with governing IRBs prior to using de-identified or coded rapid research autopsy materials.

Besides the Common Rule, another landmark regulation is the Uniformed Anatomical Gift Act (UAGA), first enacted in 1968 and subsequently revised in 1987 and 2006 [15]. The UAGA allows all or parts of the body to be gified for 3 specific purposes: 1) transplantation (or therapy), 2) research, and/or 3) education [11, 14, 15]. The Last Gift falls under the research category, and its purpose is to produce generalizable knowledge about the viral reservoir to support HIV cure research. If expressly desired by a participant, however, the Last Gift would not preclude donations for the purpose of transplantation or education. Any research using the Last Gift participant's body and donated human biological materials (cells, tissues, organs) post-mortem falls under the UAGA and follows a *gift law framework* [14]. According to the UAGA, next-of-kin/loved ones are not allowed to override the donor's decision at the time of death [14]. The UAGA further stipulates that the gift of human biological materials should be used according to the living donor's intent [14].

In 2005, the Consensus Panel on Research with the Recently Dead (CPRRD) published recommendations to guide EOL research programs in oncology [11]. The CPRRD recommended that legally required autopsies take precedence over transplantation or research [11]. Further, organ donations and research are not mutually exclusive, but should be carefully coordinated between organ procurement organizations and EOL research teams [11]. The CPRRD also stipulates the need to avoid commodifying the donor's body and the requirement to “treat the body with dignity of the once-living individual [11].” The CPRRD, therefore, prohibits selling and buying any parts of the body [11]. In contrast, the American Medical Association (AMA) adopted guidelines for commercial use of human tissues [16]. For example, human donors must provide informed consent for any commercial use of human tissues, and profits should be shared according to lawful contractual arrangements [16]. To maintain public trust in the program, the Last Gift does not permit selling and/or buying any donated human biological materials.

In the United States, the Health Insurance Portability and Accountability Act (HIPAA) of 1996 [17] governs the use of identifiable participant information for research purposes. With living individuals, HIPAA privacy laws require the completion of informed consent forms that describe how health information will be kept confidential. Accordingly, in the Last Gift, we provide a copy of the HIPAA authorization form and a Notice of Privacy Practice booklet to each participant during the informed consent process. However, for research uses of donated human biological materials post-mortem, HIPAA privacy laws become complex and may require authorizations by legally authorized representatives [14]. Depending on the specific research protocol, we recommend consulting with HIPAA officers at each institution on proposed courses of action for sharing potentially identifiable or identified health data.

Moreover, in the University of California system, donation programs must comply with the Human Anatomical Specimen and Tissue Oversight Committee (HASTOC) policy. At UCSD, this policy is managed by the Human and Animal Tissue Technology Center. It governs the procurement, inventory, use, management, transportation, storage, security, and disposition of human biological materials. The HASTOC policy came into effect following a scandal that involved the University of California Los Angeles Willed Body Program in 2004, where human remains were found to be sold illegally for profit [18]. The UCSD Human and Animal Tissue Technology Center aims to preserve public confidence in anatomical donation programs for research and education purposes. To that end, any commonly recognizable human biological materials (eg, entire organs) fall under the HASTOC policy. Specific human biological materials, however, are excluded from the HASTOC policy, including blood, urine, other bodily fluids, non-organic tissues, tissue samples, human cells, or connective tissue fragments. The HASTOC policy requires the use of a secure and centralized registry called the Digital Donor Library (DDL) and prohibits the transfer of human biological materials to third-party brokers or intermediaries. All research projects falling under the HASTOC policy are subject to quarterly reporting and regular inspections. While a cost-recovery fee for preparing materials can be applied, no UCSD employee can profit from transferring human anatomical materials.

## ETHICAL VALUES AND CONSIDERATIONS

While laws, regulations, and policies dictate what must be done, ethics seek to understand what ought to be done. The Last Gift team established baseline ethical criteria for HIV cure research at the EOL [19]. These ethical considerations include 1) protecting autonomy through informed consent, 2) avoiding exploitation and fostering altruism, 3) maintaining favorable benefit/risk criteria, 4) safeguarding against vulnerability by focusing on patients/participants, and 5) ensuring acceptance of next-of-kin/loved ones and community members [19]. The Last Gift team also prospectively documents ethical and practical lessons learned [20]. For example, we learned that PWH at the EOL wanted to exercise their right to die with dignity and compassion [20, 21].

**Figure 1.**
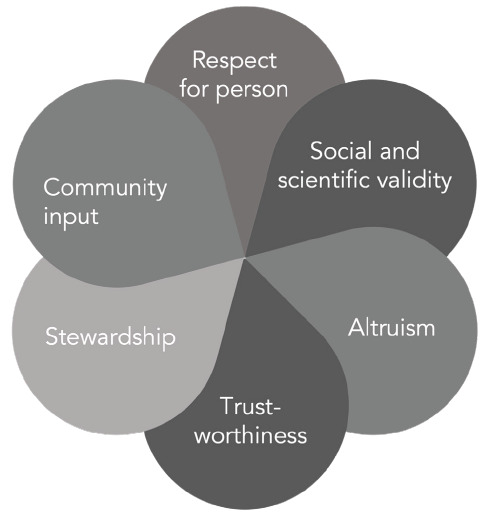
Ethical values and considerations.

While these ethical criteria have to date proven effective in governing conduct of the Last Gift program towards its initial intent, the need to prioritize additional requests for donated human biological materials has brought specific key ethical principles to the forefront. In this section, we review some of these ethical values and considerations, including respect for persons (informed consent), social value and scientific validity, altruism, trustworthiness, stewardship, and community input (Figure 1).

### Respect for persons (including informed consent).

By respect for persons, we mean respecting autonomous choices [12] and the dignity of the person. Respect for persons was described in the Belmont Report [22] and is crucial to maintaining the ethicality of donation programs such as the Last Gift. Respect for persons also entails avoiding undue pressure or coercion around decisions to donate human biomedical materials to advance scientific research of any kind [12]. Of note, in the context of cancer research at the EOL, Pentz and colleagues cautioned, that conducting concurrent studies at the EOL could be construed as disrespectful for the dying individual and their next-of-kin/loved ones [11].

All Last Gift participants complete a robust informed consent process at study entry. This implies disclosure of information, comprehension, and voluntariness [19, 23]. In addition, we follow process (or continuous) informed consent to ensure participants continue to agree to research procedures and tissue donations as they approach the EOL [19, 20, 24]. For any donation of human biological materials outside the scope of HIV cure research, the Last Gift team reviews the need for separate informed consent on a case-by-case basis. We either include a possible use of tissues directly in the Last Gift informed consent form, or follow a separate informed consent process (ie, opt-in or expressed consent). The Last Gift informed consent form has evolved over time to allow for additional possible use of tissues.

Dignity of persons is defined as “objective human thriving” [25] and is operationalized in multiple ways for both living participants and deceased donors. In the context of EOL oncology research, Pentz and colleagues advised that research teams must treat the body of the recently deceased in a dignified manner [11]. Before each rapid research autopsy, the Last Gift observes a minute of silence to honor the life of each participant and express gratitude for their gift to science and society [19]. We also strive to minimize the degree of invasiveness of study procedures necessary to generate the scientific benefits and avoid disfigurement during rapid research autopsies [11, 20].

Another way to operationalize dignity and autonomy is to respect the wishes and values of each participant [19]. As explained by Strong and Colleagues, “Anatomical gifting is akin to other dispositions upon death [26].” Following the principles of self-determination, the Last Gift relies upon stated wishes from participants expressed during the EOL decision-making process [27]. The Last Gift recommends participants to prepare advance directives, although these are not required to join the study [20]. In all cases, we view advance directives as taking precedence over research and comply with Last Gift participants' wishes contained in their advance directives [20]. We also treat each participant's EOL wishes with individualized attention. In some cases, it may be necessary to engage in the process of clarifying individual wishes at the EOL. For example, a Last Gift participant with amyotrophic lateral sclerosis (ALS) expressed the desire to have his spinal cord donated to ALS research at the EOL [19]. In other cases, we may need to consolidate donors' wishes expressed in non-Last Gift documents or advance directives, such as living wills, donor cards, or driver's licenses. It may also be necessary to know who holds power of attorney for Last Gift participants. The Last Gift team attempts to do everything possible to respect and honor each participant's wishes at the EOL.

We also observe respect for persons by paying attention to the language used in the context of research at the EOL. For example, we strongly advise against using the expression “HIV-infected subjects” to refer to Last Gift participants, who willingly express their desire to contribute to science and are considered partners in research. We also avoid using words such as “cadavers,” “harvesting tissues or organs,” and so on. We prefer the expression “providing informed consent” as opposed to “consenting,” since the decision to participate in the Last Gift study remains each participant's autonomous decision [12].

### Social value and scientific validity.

Principles of social value and scientific validity are aligned with beneficence (maximizing benefits), while minimizing potential harms [28]. In the field of organ transplantation, beneficence is oftentimes recognized as the principle of utility [12]. A related concept is that of scientific validity, because only valid results can produce scientific and societal benefits [11, 12, 29]. In the Last Gift, we operationalize social value and scientific validity by carefully reviewing each concurrent donation request. Proposals must address important scientific research questions and must offer to utilize donated human biological materials in an efficient manner to generate high-impact generalizable knowledge.

### Altruism.

EOL HIV cure research relies on altruism as a cornerstone principle, which underpins any donation of human biological materials [30]. With the possibility of allocating human biological materials for scientific aims beyond HIV cure research, altruism is brought into sharp focus. In relation to the ethics of organ donations at the EOL, Rosenbaum employed the expression “altruism in extremis” to describe donors' deep manifestation of generosity, selflessness, and terminal kindness [31]. Our empirical research with Last Gift participants revealed that altruism was embedded within the context of community, scientific advancement, love for humanity, and moral obligation [4]. Altruism in death and existentialism, a deep form of activism, were signifi-cant themes in our interviews with Last Gift participants and their next-of-kin/loved ones [4, 32, 33]. Anecdotally, we learned that altruism in the Last Gift tends to be directed toward HIV/AIDS. While Last Gift participants dislike their terminal or co-existing illnesses, they hate their HIV – and often having witnessed the ravaging effects of AIDS when the condition was untreatable – wish to contribute at the EOL to ending this epidemic [4, 5].

### Trustworthiness.

Trustworthiness has been defined as the “confidence in and reliance on others to act competently and in accord with ethical principles and legal and regulatory standards [12].” Trustworthiness is imperative to maintaining public confidence in research at the EOL. Having a prioritization process in place for the use of donated human biological materials can help promote trust. A recent report from the National Academies of Sciences, Engineering, and Medicine indicated there can be several types of trust, including trust in the research teams to act diligently and competently, as well as trust in next-of-kin/loved ones to help honor the participants' wishes at the EOL [12]. For donation programs such as the Last Gift3, trust is paramount, but it can also be fragile, and must guide our prioritization decisions [12].

### Stewardship.

By stewardship, we mean honoring our duties to help fulfill participants' wishes at the EOL [34]. One way to act as good stewards of donated biological materials is to help avoid potential conflicts between multiple concurrent research aims and/or studies and between Last Gift participants and their next-of-kin/loved ones [33, 35]. For example, the Last Gift aims to primarily advance HIV cure research. We recognize that this may represent an internal conflict for participants with co-morbidities at the EOL wanting to contribute to other scientific causes [19]. We have open and transparent conversations about any potential conflict of participation. To date, the Last Gift has remained observational, and has not yet involved interventional research at the EOL [36]. Should we cross that bridge, we will need to review on a case-by-case basis and proactively address any potential conflict and rely on inclusion/exclusion criteria of respective clinical research protocols to assess whether participation in individual studies or anatomical gifts would be advisable [19, 37].

### Community input.

Community input and advocacy has been a critical component of the Last Gift since its inception [19], even before US National Institutes of Health grant reviewers priori-tized community participation as an integral part of research [24]. We hold that primary community stakeholders – especially PWH – should have a say in prioritizing the use of donated human biological materials. Therefore, we engage in periodic dialogues around proposed research concepts. These conversations have led to the creation of the present community-driven prioritization framework for the use of donated human biological materials.

## COMMUNITY-DRIVEN PRIORITIZATION FRAMEWORK FOR USE OF DONATED HUMAN BIOLOGICAL MATERIALS

**Figure 2.**
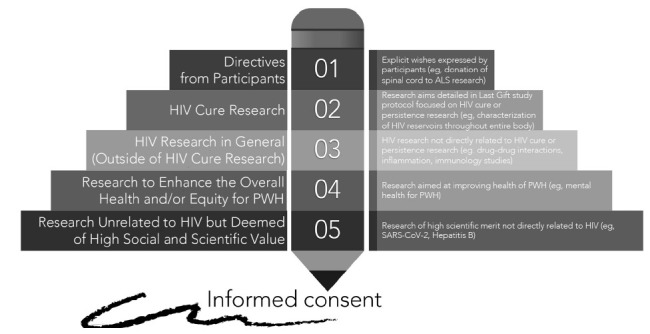
Community-driven framework to prioritize use of donated human biological materials in the context of HIV cure-related research at the end of life.

The prioritization framework (Figure 2) presented in this manuscript was the original concept of Thomas Villa (second author), a community member who advises on the Last Gift study. The prioritization framework was refined in collaboration with the AntiViral Research Center Community Advisory Board and the Last Gift team. This prioritization framework can serve as a model for similar rapid research autopsy programs at the EOL or tissue repositories.

When we survey community members about their views regarding requests for donated human biological materials in the Last Gift, we pose various questions to understand where they draw their line in terms of the acceptable use of biospecimens. For all non-Last Gift-related requests, we also use concept sheets from the research study in question to help evaluate the scientific merit and community acceptability. Importantly, we try to avoid the Last Gift team from being overwhelmed by concurrent external studies or concepts and pulled away from Last Gift's priorities of advancing HIV cure research.

### Priority 1: Clear directives from participants and HIV cure research.

We first prioritize directives from Last Gift participants. A paradigmatic example included the donation of a spinal cord to the field of ALS research as described above.

The research aims detailed in the Last Gift study protocol (“The Last Gift: Development of an End-of-Life Translational Model to Characterize the HIV Reservoir”) is also a top priority, together with any directive from participants. Participants provide informed consent for the collection of blood draws and biospecimens until the EOL. Participants also provide informed consent for a rapid research autopsy shortly following death to characterize HIV reservoirs throughout the entire body.

Research examples in this category include characterizing the HIV reservoir size, activity, and diversity using digital droplet polymerase chain reaction and single genome sequencing techniques, as well as applying single-cell sequencing assays to identify cell determinants relevant to the persistence of HIV infection [9]. Methods have included single nuclei ribonucleic acid sequencing and single-cell sequencing assay for transposase-accessible chromatin technologies, bioinformatic approaches, and validation studies. Related to brain tissues, for example, we have performed analyses in microglia and tissue-resident macrophages [24]. Laboratory analyses help reveal the genetics and epigenetics regulation of HIV reservoir dynamics throughout the entire human body.

The Last Gift has also established a robust and dynamic network of HIV cure research collaborators. For instance, external collaborations allow the definition of HIV reservoirs in the central nervous system (CNS) – including neuronal, glial, myeloid, and lymphoid cells from CNS tissues, and measurements of persistent HIV infection using various assays. Another Last Gift collaboration led to findings regarding evidence of resistance susceptibility profiles compartmentalized in tissues, such as the brain, that could be the source of viral rebound and lead to therapeutic failure of broadly neutralizing antibodies [38]. Several external Last Gift collaborations are currently underway to help characterize HIV reservoirs throughout the entire human body, including the gut, spleen, kidneys, lymphoid tissues, and other body compartments. These analyses combine various disciplines, including molecular biology, phylodynamic analyses, immunology, virology, and biostatistics, among others.

### Priority 2: HIV research in general (separate from HIV cure research).

We would then priori-tize HIV research in general (separate from HIV cure research). The main reason is that Last Gift participation is primarily motivated by HIV-specific altruism, as described above [4, 30]. This category requires a case-by-case review of whether to include the possible use of human tissues directly in the Last Gift informed consent form or whether to request separate opt-in informed consent from study participants. Examples of possible projects could include understanding how chronic HIV may affect cardiovascular health and disease for PWH, cancer among PWH, or how HIV interacts with other non-communicable diseases exacerbated by HIV. Additional projects could include pharmacological methods to understand the distribution of HIV antiretrovirals throughout the entire body, studies aimed at understanding drug-drug interactions and inflammation, or immunology studies not directly linked to HIV cure research.

### Priority 3: Research to enhance the overall health and/or equity for PWH.

Our next priority would be research protocols aimed at enhancing health and health equity for PWH. Although causes of HIV-associated morbidity and mortality for PWH blend with those of the general population [3], PWH must confront challenges associated with the onset of illnesses much earlier (ie, 10 years earlier) than people without HIV [2], Further, there remain important racial and ethnic disparities along the HIV care continuum in the United States. These disparities worsen alongside aging, with worse morbidity and mortality outcomes for African Americans/Blacks and Hispanics/Latinos [39, 40]. PWH also experience somatic symptoms associated with the longevity of HIV, such as fatigue, pain, insomnia, and decreased memory and concentration [3, 41, 42]. These health issues are exacerbated by, and occur in tandem with, social (eg, ageism, stigma, social isolation, erosion of social support), mental health (eg, depression, anxiety), and emotional challenges [2, 3, 43]. Any research project aimed at enhancing the overall health and equity for PWH would fall in this category and would require separate opt-in informed consent.

As an example, we describe an external bone donation tissue request. In 2022, the Last Gift was approached by a small company wanting to develop novel ways to measure bone health in PWH, using touch instead of imaging. The company was interested to test a tactile bone measurement device and inquired whether the Last Gift would be interested to collaborate. The study would require the extraction of entire bones to perform mechanical experiments, a process more invasive than our usual procedures and subject to HASTOC oversight (since bones are considered recognizable human parts). One of the study rationales given was that PWH have lower bone strength than the general population because both HIV and treatments weaken bones. The scientific rationale of the study was to improve ways to measure bone health in PWH. These measurements would help improve the management of bone strength/health and help increase mobility and quality of life for PWH as they age. Importantly, this study was federally funded, and the company had no direct financial benefit from this small pilot study.

The Last Gift team evaluated this proposal carefully and invited community members to comment on a series of meetings. Several considerations were given, including recognizing the importance of the research question, the need for separate informed consent, and to ensure the highest potential future clinical significance for PWH. There would also be a need to clearly explain the potential scientific merits of this proposal to the community, including explaining why specific human bones (eg, tibia, hip, spine, wrist) would be needed for research. Further, because the device is proposed to be evaluated in PWH, it should ultimately benefit PWH.

Community members were divided on this proposal. Some argued that PWH would want their donations to benefit science as much as possible without wanting to know all the details of the autopsy procedure, which might be overwhelming for some. Other community members were reluctant to give donations to companies that might eventually make a profit and questioned whether the community would be accepting of this research. They did not want PWH to feel pressured to participate and stressed the need for clear, effective communication with next-of-kin/loved ones. Concern was expressed that the bone research supplement could lead to misconceptions about the Last Gift in the community and that it would pull the Last Gift research staff's attention away from the initial intent (scientific drift). Logistically, the bone supplement would fall under the UCSD HASTOC policy (described above), which could mean higher costs, specialized staff needed for rapid research autopsies, and more onerous regulatory and administrative steps for the Last Gift team. The Last Gift team has decided to move forward extremely cautiously with this supplement after multiple deliberations.

### Priority 4: Research unrelated to HIV but deemed of high social and scientific value.

The next category involves proposed research unrelated to HIV but deemed of high social and scientific value. This category would require separate opt-in informed consent from study participants, and co-enrollment is possible. A recent example includes a research supplement related to COVID-19 infection. Our team is implementing a study to evaluate factors that might influence the SARSCoV-2 viral and immunity dynamics and host responses with and without prior vaccination and immune responses after vaccination. Additional examples may include research aimed at identifying the underlying genetic and immune mechanisms driving the severity of infectious diseases or donation of liver tissues to the field of Hepatitis B (HBV) cure research to characterize HBV reservoirs in PWH living with HBV.

### Framework limitations.

We recognize a major limitation to our prioritization framework for the use of donated human biological materials in the context of HIV cure-related research at the EOL. The framework was developed in the context of the Last Gift, implemented at one infectious diseases research center in the United States.

## CONCLUSIONS

The sustainability of EOL research programs relies on community trust and ethical decision-making. In this manuscript, we propose a prioritization framework for the use of donated human biological materials from altruistic PWH, using the Last Gift study as a case study. The key take-away messages from our community advisors are to maintain the original scope of the project, to prioritize the research with the greatest social and scientific value, and to be intentional about sharing tissues with possible collaborators. Establishing an accepted prioritization framework to help adjudicate requests for precious biological specimens (eg, cells, tissues) can help propel scientific discoveries forward. Our research community has an unprecedented duty to honor PWH's extraordinary gift to humanity.

## SUPPLEMENTARY DATA

The UCSD Last Gift rapid research autopsy protocol is published [8]. The authors can make additional materials available upon request, such as Standard Operating Procedures, informed consent forms, and other documentation. Please contact Sara Gianella (gianella@health.ucsd.edu) or Davey Smith (d13smith@ucsd.edu) to request the most recent version of these materials.

## References

[1] Palella FJ, Jr., Delaney KM, Moorman AC, Loveless MO, Fuhrer J, Satten GA, Aschman DJ, Holmberg SD. Declining morbidity and mortality among patients with advanced human immunodeficiency virus infection. HIV Outpatient Study Investigators. *N Engl J Med.* 1998;338(13):853–60. doi: 10.1056/NEJM199803263381301. PubMed PMID: 9516219. 9516219

[2] Brown B, Marg L, Cabral A, Didero M, Christensen C, Taylor J, Subica A. Community-Driven Health Priorities for Healthy Aging With HIV. *J Assoc Nurses AIDS Care.* 2019;30(1):119–28. doi: 10.1097/JNC.0000000000000042. PubMed PMID: 30586089. 30586089

[3] Goodkin K, Kompella S, Kendell SF. End-of-Life Care and Bereavement Issues in Human Immunodeficiency Virus-AIDS. *Nurs Clin North Am.* 2018;53(1):123–35. doi: 10.1016/j.cnur.2017.10.010. PubMed PMID: 29362056; PMCID: PMC5837059. 29362056 PMC5837059

[4] Perry KE, Dubé K, Concha-Garcia S, Patel H, Kaytes A, Taylor J, Javadi SS, Mathur K, Lo M, Brown B, Sauceda JA, Wohl DA, Little S, Hendrickx S, Rawlings SA, Smith DM, Gianella S. “My Death Will Not [Be] in Vain”: Testimonials from Last Gift Rapid Research Autopsy Study Participants Living with HIV at the End of Life. *AIDS Res Hum Retroviruses*. 2020;36(12):1071–82. Epub 2020 06 24. doi: 10.1089/AID.2020.0020. PubMed PMID: 32449625; PMCID: PMC7703253. 32449625 PMC7703253

[5] Gianella S, Taylor J, Brown TR, Kaytes A, Achim CL, Moore DJ, Little SJ, Ellis RJ, Smith DM. Can research at the end of life be a useful tool to advance HIV cure? *AIDS*. 2017;31(1):1–4. doi: 10.1097/QAD.0000000000001300. PubMed PMID: 27755112; PMCID: PMC5137789. 27755112 PMC5137789

[6] Lessard D, Dubé K, Bilodeau M, Keeler P, Margolese S, Rosenes R, Sinyavskaya L, Durand M, Benko E, Kovacs C, Guerlotte C, Tharao W, Arnold K, Masching R, Taylor D, Sousa J, Ostrowski M, Taylor J, Kaytes A, Smith D, Gianella S, Chomont N, Angel JB, Routy JP, Cohen EA, Lebouche B, Costiniuk CT. Willingness of Older Canadians with HIV to Participate in HIV Cure Research Near and After the End of Life: A Mixed-Method Study. *AIDS Res Hum Retroviruses.* 2022;38(8):670–82. Epub 2022 07 28. doi: 10.1089/AID.2022.0006. PubMed PMID: 35778845; PMCID: PMC9483839. 35778845 PMC9483839

[7] Prakash K, Gianella S, Dubé K, Taylor J, Lee G, Smith DM. Willingness to participate in HIV research at the end of life (EOL). *PLoS One*. 2018;13(7):e0199670. Epub 2018 07 23. doi: 10.1371/journal.pone.0199670. PubMed PMID: 30036365; PMCID: PMC6056048. 30036365 PMC6056048

[8] Rawlings SA, Layman L, Smith D, Scott B, Ignacio C, Porrachia M, Concha-Garcia S, Hendrickx S, Kaytes A, Taylor J, Gianella S. Performing rapid autopsy for the interrogation of HIV reservoirs. *AIDS*. 2020;34(7):1089–92. doi: 10.1097/QAD.0000000000002546. PubMed PMID: 32287073; PMCID: PMC7780881. 32287073 PMC7780881

[9] Chaillon A, Gianella S, Dellicour S, Rawlings SA, Schlub TE, De Oliveira MF, Ignacio C, Porrachia M, Vrancken B, Smith DM. HIV persists throughout deep tissues with repopulation from multiple anatomical sources. *J Clin Invest.* 2020;130(4):1699–712. doi: 10.1172/JCI134815. PubMed PMID: 31910162; PMCID: PMC7108926. 31910162 PMC7108926

[10] Rawlings SA, Chaillon A, Smith D, Gianella S. Scale up rapid research autopsies for tissue immunology. *Nature*. 2021;595(7867):352. doi: 10.1038/d41586-021-01887-y. PubMed PMID: 34257439. PMC11140983 34257439

[11] Pentz RD, Cohen CB, Wicclair M, DeVita MA, Flamm AL, Youngner SJ, Hamric AB, McCabe MS, Glover JJ, Kittiko WJ, Kinlaw K, Keller J, Asch A, Kavanagh JJ, Arap W. Ethics guidelines for research with the recently dead. *Nat Med.* 2005;11(11):1145–9. doi: 10.1038/nm1105-1145. PubMed PMID: 16270065. 16270065

[12] Opportunities for Organ Donor Intervention Research [Internet]. NASEM; 2020. Available from: https://nap.nationalacademies.org/catalog/24884/opportunities-for-organ-donor-intervention-research-saving-lives-by-improving.

[13] Code of Federal Regulations Title 45 Part 46 Protection of Human Subjects [Internet]. US Department of Health and Human Services; 2014. Available from: https://www.hhs.gov/ohrp/regulations-and-policy/regulations/45-cfr-46/index.html.

[14] Glazier AK, Heffernan KG, Rodrigue JR. A Framework for Conducting Deceased Donor Research in the United States. *Transplantation*. 2015;99(11):2252–7. doi: 10.1097/TP.0000000000000841. PubMed PMID: 26244717. 26244717

[15] Revised Uniform Anatomical Gift Act [Internet]. National Conference of Commissioners on Uniform State Laws; 2006 1-60]. Available from: https://wcmea.com/wp-content/uploads/2020/01/Uniform-Anatomical-Gift-Act.pdf.

[16] Commercial Use of Human Biological Materials: American Medical Association. Available from: https://code-medical-ethics.ama-assn.org/ethics-opinions/commercial-use-human-biological-materials#:~:text=Human%20biological%20materials%20and%20their,accordance%20with%20lawful%20contractual%20agreements.

[17] The HIPAA Privacy Rule [Internet]. Department of Health and Human Services; [updated March 31, 2022; cited 2023 March]. Available from: https://www.hhs.gov/hipaa/for-professionals/privacy/index.html.

[18] UCLA suspends its Willed Body Program [Internet]. CNN.com; Wednesday, March 10, 2004. Available from: https://edition.cnn.com/2004/LAW/03/09/ucla.cadaver.suit/index.html.

[19] Dubé K, Gianella S, Concha-Garcia S, Little SJ, Kaytes A, Taylor J, Mathur K, Javadi S, Nathan A, Patel H, Luter S, Philpott-Jones S, Brown B, Smith D. Ethical considerations for HIV cure-related research at the end of life. *BMC Med Ethics*. 2018;19(1):83. Epub 2018 10 20. doi: 10.1186/s12910-018-0321-2. PubMed PMID: 30342507; PMCID: PMC6196016. 30342507 PMC6196016

[20] Kanazawa J, Rawlings SA, Hendrickx S, Gianella S, Concha-Garcia S, Taylor J, Kaytes A, Patel H, Ndukwe S, Little SJ, Smith D, Dubé K. Lessons learned from the Last Gift study: ethical and practical challenges faced while conducting HIV cure-related research at the end of life. *J Med Ethics*. 2023;49(5):305–10. Epub 2022 06 22. doi: 10.1136/medethics-2021-107512. PubMed PMID: 35732421; PMCID: PMC9772357. 35732421 PMC9772357

[21] Danielson MM, Dubé K. Michael's Testimonial. *Ann Intern Med*. 2018;169(5):349. doi: 10.7326/M18-1719. PubMed PMID: 30178018. 30178018

[22] Ethical Principles and Guidelines for the Protection of Human Subjects of Research. The National Commission for the Protection of Human Subjects of Biomedical and Behavioral Research [Internet]. Department of Health, Education, and Welfare; April 18, 1979 [Notice of Report for Public Comment]. Available from: https://www.hhs.gov/ohrp/regulations-and-policy/belmont-report/read-the-belmont-report/index.html.

[23] Gordon EJ. Informed consent for living donation: a review of key empirical studies, ethical challenges and future research. *Am J Transplant.* 2012;12(9):2273–80. Epub 2012 05 17. doi: 10.1111/j.1600-6143.2012.04102.x. PubMed PMID: 22594620. 22594620

[24] Riggs PK, Chaillon A, Jiang G, Letendre SL, Tang Y, Taylor J, Kaytes A, Smith DM, Dubé K, Gianella S. Lessons for Understanding Central Nervous System HIV Reservoirs from the Last Gift Program. *Curr HIV/AIDS Rep.* 2022;19(6):566–79. Epub 2022 10 19. doi: 10.1007/s11904-022-00628-8. PubMed PMID: 36260191; PMCID: PMC9580451. 36260191 PMC9580451

[25] Foster C. Dignity and the ownership and use of body parts. *Camb Q Healthc Ethics.* 2014;23(4):417–30. Epub 2014 07 17. doi: 10.1017/S0963180114000097. PubMed PMID: 25032711. 25032711

[26] Strong CW, Shafer T. Donation of bodily material for medicine and research. *BMJ*. 2011;343:d6839. Epub 2011 10 26. doi: 10.1136/bmj.d6839. PubMed PMID: 22031927. 22031927

[27] Freeman RB, Bernat JL. Ethical issues in organ transplantation. *Prog Cardiovasc Dis.* 2012;55(3):282–9. doi: 10.1016/j.pcad.2012.08.005. PubMed PMID: 23217432. 23217432

[28] Beauchamp TL CJ. Principles of Biomedical Ethics. 8 ed: Oxford University Press; 2019. 512 p.

[29] Joffe S, Miller FG. Bench to bedside: mapping the moral terrain of clinical research. *Hastings Cent Rep.* 2008;38(2):30–42. doi: 10.1353/hcr.2008.0019. PubMed PMID: 18457227. 18457227

[30] Dubé K, Perry KE, Mathur K, Lo M, Javadi SS, Patel H, Concha-Garcia S, Taylor J, Kaytes A, Dee L, Campbell D, Kanazawa J, Smith D, Gianella S, Auerbach JD, Saberi P, Sauceda JA. Altruism: Scoping review of the literature and future directions for HIV cure-related research. *J Virus Erad.* 2020;6(4):100008. Epub 2020 08 25. doi: 10.1016/j.jve.2020.100008. PubMed PMID: 33294210; PMCID: PMC7695811. 33294210 PMC7695811

[31] Rosenbaum L. Altruism in Extremis - The Evolving Ethics of Organ Donation. *N Engl J Med.* 2020;382(6):493–6. doi: 10.1056/NEJMp2000048. PubMed PMID: 32023370. 32023370

[32] Dubé K, Patel H, Concha-Garcia S, Perry KE, Mathur K, Javadi SS, Taylor J, Kaytes A, Brown B, Sauceda JA, Little S, Hendrickx S, Rawlings SA, Smith DM, Gianella S. Perceptions of Next-of-Kin/Loved Ones About Last Gift Rapid Research Autopsy Study Enrolling People with HIV/AIDS at the End of Life: A Qualitative Interview Study. *AIDS Res Hum Retroviruses.* 2020;36(12):1033–46. Epub 2020 06 25. doi: 10.1089/AID.2020.0025. PubMed PMID: 32449624; PMCID: PMC7703245. 32449624 PMC7703245

[33] Javadi SS, Mathur K, Concha-Garcia S, Patel H, Perry KE, Lo M, Taylor J, Kaytes A, Little S, Gianella S, Smith D, Dubé K. Attitudes and perceptions of next-of-kin/loved ones toward end-of-life HIV cure-related research: A qualitative focus group study in Southern California. *PLoS One*. 2021;16(5):e0250882. Epub 2021 05 07. doi: 10.1371/journal.pone.0250882. PubMed PMID: 33961653; PMCID: PMC8104928. 33961653 PMC8104928

[34] Perry KE, Taylor J, Patel H, Javadi SS, Mathur K, Kaytes A, Concha-Garcia S, Little S, Smith D, Gianella S, Dubé K. “[It] is now my responsibility to fulfill that wish:” Clinical and rapid autopsy staff members' experiences and perceptions of HIV reservoir research at the end of life. *PLoS One.* 2020;15(11):e0242420. Epub 2020 11 18. doi: 10.1371/journal.pone.0242420. PubMed PMID: 33206710; PMCID: PMC7673534. 33206710 PMC7673534

[35] Wendler D, Dickert N. The consent process for cadaveric organ procurement: how does it work? How can it be improved? *JAMA*. 2001;285(3):329–33. doi: 10.1001/jama.285.3.329. PubMed PMID: 11176844. 11176844

[36] Kanazawa J, Gianella S, Concha-Garcia S, Taylor J, Kaytes A, Christensen C, Patel H, Ndukwe S, Rawlings S, Hendrickx S, Little S, Brown B, Smith D, Dubé K. Ethical and practical considerations for interventional HIV cure-related research at the end-of-life: A qualitative study with key stakeholders in the United States. *PLoS One.* 2021;16(7):e0254148. Epub 2021 07 16. doi: 10.1371/journal.pone.0254148. PubMed PMID: 34270612; PMCID: PMC8284787. 34270612 PMC8284787

[37] Pope TM. Legal briefing: organ donation and allocation. *J Clin Ethics.* 2010;21(3):243–63. PubMed PMID: 21089996. 21089996

[38] Wang C, Schlub TE, Yu WH, Tan CS, Stefic K, Gianella S, Smith DM, Lauffenburger DA, Chaillon A, Julg B. Landscape of Human Immunodeficiency Virus Neutralization Susceptibilities Across Tissue Reservoirs. *Clin Infect Dis.* 2022;75(8):1342–50. doi: 10.1093/cid/ciac164. PubMed PMID: 35234862; PMCID: PMC9555844. 35234862 PMC9555844

[39] Lang ME, Bird CE. Understanding and Addressing the Common Roots of Racial Health Disparities: The Case of Cardiovascular Disease and HIV/AIDS in African Americans. *Health Matrix Clevel.* 2015;25:109–38. PubMed PMID: 29485842. 29485842

[40] Sangaramoorthy T, Jamison A, Dyer T. Older African Americans and the HIV Care Continuum: A Systematic Review of the Literature, 2003-2018. *AIDS Behav.* 2019;23(4):973-83. doi: 10.1007/s10461-018-2354-4. PubMed PMID: 30519903; PMCID: PMC6459701. 30519903 PMC6459701

[41] Goodkin K, Heckman T, Siegel K, Linsk N, Khamis I, Lee D, Lecusay R, Poindexter CC, Mason SJ, Suarez P, Eisdorfer C. “Putting a face” on HIV infection/AIDS in older adults: a psychosocial context. *J Acquir Immune Defic Syndr*. 2003;33 Suppl 2:S171–84. PubMed PMID: 12853867. 12853867

[42] Rhoades N, Mendoza N, Jankeel A, Sureshchandra S, Alvarez AD, Doratt B, Heidari O, Hagan R, Brown B, Scheibel S, Marbley T, Taylor J, Messaoudi I. Altered Immunity and Microbial Dysbiosis in Aged Individuals With Long-Term Controlled HIV Infection. *Front Immunol.* 2019;10:463. Epub 2019 03 12. doi: 10.3389/fimmu.2019.00463. PubMed PMID: 30915086; PMCID: PMC6423162. 30915086 PMC6423162

[43] Marg LZ, Heidari O, Taylor J, Marbley C, Scheibel S, Hagan R, Messaoudi I, Mendoza N, Brown B. A Multidimensional Assessment of Successful Aging Among Older People Living with HIV in Palm Springs, California. *AIDS Res Hum Retroviruses*. 2019;35(11-12):1174–80. Epub 2019 09 25. doi: 10.1089/AID.2019.0048. PubMed PMID: 31441322. 31441322

